# Tools for Assessing Cardiovascular Disease Risk Factors in Underserved Young Adult Populations: A Systematic Review

**DOI:** 10.3390/ijerph182413305

**Published:** 2021-12-17

**Authors:** Audrey A. Opoku-Acheampong, Richard R. Rosenkranz, Koushik Adhikari, Nancy Muturi, Cindy Logan, Tandalayo Kidd

**Affiliations:** 1 Department of Food, Nutrition, Dietetics, and Health, Kansas State University, Manhattan, KS 66506, USA; abampoe@ksu.edu (A.A.O.-A.); ricardo@ksu.edu (R.R.R.); 2 Department of Food Science and Technology, College of Agricultural & Environmental Sciences, University of Georgia, Griffin, GA 30223, USA; koushik7@uga.edu; 3 A. Q. Miller School of Journalism and Mass Communication, Kansas State University, Manhattan, KS 66506, USA; nmuturi@ksu.edu; 4 Academic Services, Hale Library, Kansas State University, Manhattan, KS 66506, USA; clogan@ksu.edu

**Keywords:** non-clinical, risk assessment, underserved, young adult

## Abstract

Cardiovascular disease (CVD, i.e., disease of the heart and blood vessels) is a major cause of death globally. Current assessment tools use either clinical or non-clinical factors alone or in combination to assess CVD risk. The aim of this review was to critically appraise, compare, and summarize existing non-clinically based tools for assessing CVD risk factors in underserved young adult (18–34-year-old) populations. Two online electronic databases—PubMed and Scopus—were searched to identify existing risk assessment tools, using a combination of CVD-related keywords. The search was limited to articles available in English only and published between January 2008 and January 2019. Of the 10,383 studies initially identified, 67 were eligible. In total, 5 out of the 67 articles assessed CVD risk in underserved young adult populations. A total of 21 distinct CVD risk assessment tools were identified; six of these did not require clinical or laboratory data in their estimation (i.e., non-clinical). The main non-clinically based tools identified were the Heart Disease Fact Questionnaire, the Health Beliefs Related to CVD-Perception measure, the Healthy Eating Opinion Survey, the Perception of Risk of Heart Disease Scale, and the WHO STEPwise approach to chronic disease factor surveillance (i.e., the STEPS instrument).

## 1. Introduction

Between 2017 and 2018, approximately 42% of United States adults aged ≥ 20 years were obese, with approximately 9% falling in the class 3 (extreme or severe) obesity category [1]. Obesity is a risk factor for cardiovascular disease (CVD, i.e., disease of the heart and blood vessels), diabetes, and related health conditions, such as coronary heart disease (CHD), heart failure, and stroke [2]. CHD is the narrowing of the inner walls of the blood vessels that transport blood to the heart (arteries) due to a build-up of a waxy substance (plaque) and is the number one cause (45.1%) of CVD-related deaths in the U.S., with stroke (16.5%) and high blood pressure (9.1%) being the next two highest [3,4]. Globally, CVD is the leading cause of death, and in the U.S., it is estimated that one in every three deaths is attributable to CVD [3,5]. It is further projected that by 2030, about 23.6 million deaths will result from CVD events [3].

Cardiovascular risk assessment is necessary for effective CVD prevention intervention, especially in high-risk individuals [6]. Early screening in youth is encouraged to prevent cardiovascular events in adulthood as current evidence suggests that CVD risk factors are even present in adolescence [7]. To date, both paper-based and electronic risk scores have been clinically applied to estimate absolute risks using patients’ data and published equations. Current risk assessment tools either use family medical history (FH) alone or in combination with clinical factors (e.g., cholesterol level, blood pressure, and glucose level) or non-clinical factors (e.g., gender, race, weight, height, dietary and physical activity) to assess disease risk. However, there are some limitations to these tools, including non-representative or historically dated populations, limited ethnic representation, and narrowly defined and unreliable endpoints [8].

CVD risk scores or algorithms (equations) were originally used in disease diagnoses by health care practitioners, but they could also be used in public health settings as health promotion tools [9]. A non-clinical- or non-laboratory-based assessment tool is particularly useful and a cost-effective option in limited-resource settings, where access to clinical samples or factors may be challenging [10,11]. Further, in resource-deficient, ethnic minority and/or underserved communities, where members have less or no health insurance coverage, higher cost barriers to health care access, and poor self-rated general health, disease diagnoses with a family health history or clinical tool may be elusive. Thus, until an illness becomes life-threatening, it may be difficult to detect it and even attempt to control it. [12,13]. Previous reviews have identified and evaluated the accuracy of available tools to assess cardiovascular risk factors in general adult populations but not in young adults [14,15,16,17,18]. For example, Gaziano et al. [18] compared non-blood-based and blood-based total cardiovascular risk scores in seven countries and concluded that in terms of performance, both types of risk scores equally predicted risk in the cohorts studied. Chamnan et al. [17] also evaluated the performance of available CVD risk scores used among patients with diabetes and found differences between risk scores originally developed in these individuals compared to those developed in the general population. A recent review by Sacramento et al. [14] described the available methods and assessment tools for the population at high risk of CVD. However, a summary of available CVD risk assessment tools, specifically in young adults, is lacking.

Thus, the primary aim of this review is to critically appraise, compare, and summarize existing non-clinically based tools for assessing CVD risk factors in young adult populations, particularly underserved young adults. Specifically, the objectives are to summarize: (1) the instruments/questionnaires used to assess lifestyle CVD risk factors (i.e., smoking, nutritional behaviors, alcohol use, and physical activity, hereafter referred to as SNAP risk factors) in young adult populations (18–34 years old); (2) the existing instruments to assess risk factors in young adults from underserved populations.

## 2. Materials and Methods

### 2.1. Search Strategy

To avoid creating a redundant review, an initial search for systematic reviews on “cardiovascular disease risk assessment tools” was performed in the Cochrane Database of Systematic Reviews. This yielded 25 Cochrane reviews. A literature search was performed by one of the researchers (A.A.O.A.) in PubMed and Scopus between May and June 2019 to identify studies published in the English language between January 2008 and January 2019. The period from 2008 to 2019 was selected to build upon the evidence obtained from a previous systematic review [15], which spanned studies published from 1 January 1999 to 24 February 2009. The search strategy and keywords used followed guidelines outlined in the COSMIN (Consensus-based Standards for the selection of health status Measurement Instruments) manual for Systematic Reviews of Patient-Reported Outcome Measures (PROMs) [19] as well as those used by Matheny et al. [15]. PubMed and MeSH on Demand version 2.0 were also used to generate a concept table (Table 1) and search terms for the search. A comprehensive search strategy used in PubMed is presented in (Table S1). Reference lists from retrieved full-text articles were also examined for any other potential studies.

### 2.2. Eligibility Criteria

The citations and abstracts from all the retrieved studies were downloaded to Rayyan for Systematic Reviews (a free web/mobile application; https://www.rayyan.ai/ (accessed on 30 June 2019)) [20] and RefWorks Citation Manager (version 2.1.0.1, Ex Libris, Chicago, IL, USA). Duplicate articles were then deleted. The selection for inclusion into the review was performed by first screening the titles and abstracts and then reviewing the full text of the articles against the inclusion/exclusion criteria outlined in Table 2.

### 2.3. Data Extraction

A data extraction form (Figure S1) was developed to extract the following information: study details (authors, year, country of origin, and study design (e.g., cross-sectional, cohort, etc.)), participants (study population, characteristics, and setting), CVD risk factors (smoking, nutrition, alcohol intake, and physical activity) assessed, CVD risk assessment tools used, and study results/findings. Eligible articles were then classified into two groups based on the two objectives of this review: articles related to CVD risk assessment in the general young adult population, and articles concerning assessment in underserved young adults.

### 2.4. Analyses of Results

The results were summarized by descriptive statistics. A quantitative synthesis of the identified tools was beyond the scope of this review.

## 3. Results

**Figure 1:**
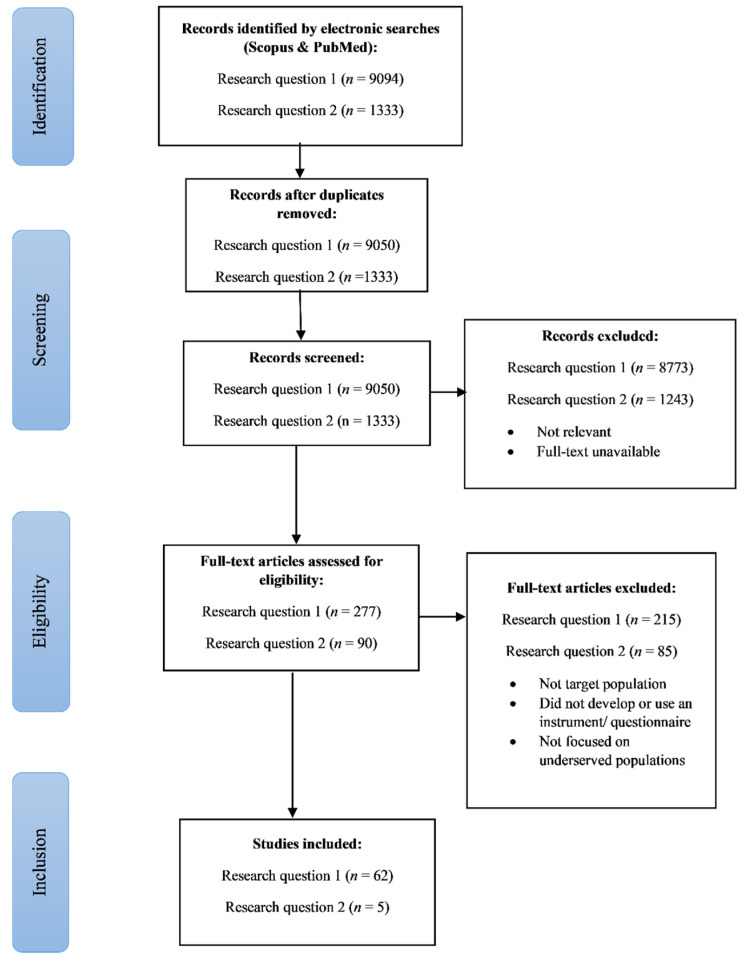
PRISMA flow chart of electronic database search.

The PRISMA (Preferred Reporting Items for Systematic Reviews and Meta-Analyses) flow chart of the study selection process is presented in Figure 1. The initial search identified a total of 10,383 studies, which was then further limited to full text and abstracts, narrowing the total down to 367 articles. Overall, 67 articles were eligible, 5 of which assessed CVD risk in underserved young adult populations (Figure 1).

### 3.1. Studies Assessing CVD Risks in the General Young Adult Population

Table 3 presents a summary of the included articles that used non-clinical tools to assess CVD risk factors in the general young adult population, including studies that included young adults only as a sub-group analysis of a broader age range of adults.

#### 3.1.1. Study Designs and Populations

**Figure 2:**
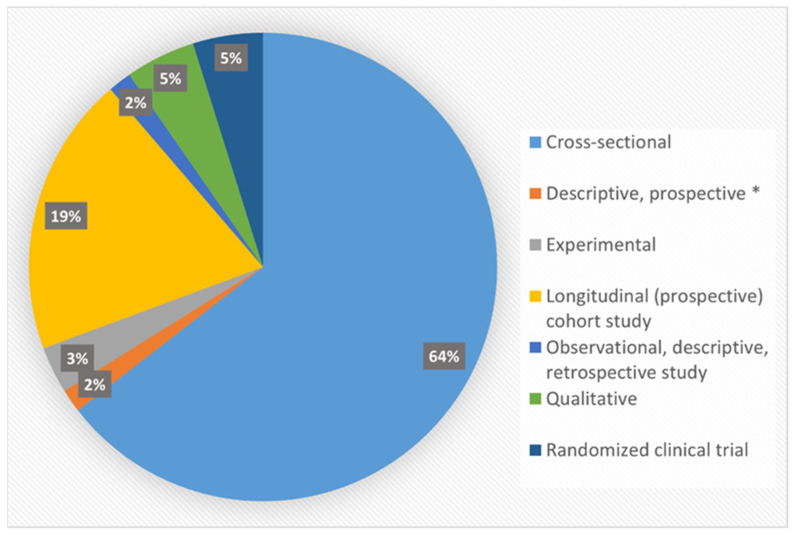
Distribution of included studies by study designs (Note: * Qualitative study).

Most (*n* = 40, 64%) of the included studies were cross-sectional in design (Figure 2). The study populations comprised healthy individuals from both general adult and young adult populations. Almost one-third (*n* = 19, 30.6%) of the included studies’ participants were within the 18–44 and 20–49 years age ranges.

#### 3.1.2. CVD Outcomes

The assessed CVD outcomes included the prevalence of CVD risk factors (e.g., total cholesterol, high-density lipoprotein cholesterol, and low-density lipoprotein cholesterol), hypertension, FH of CVD/CHD, the presence of metabolic syndrome, diabetes, CHD risk, ideal cardiovascular health (ICH) index, the perception of heart disease risk, awareness of lifestyle risk factors, and knowledge of CHD. About half of the studies (*n* = 30, 48%) assessed at least two SNAP risk factors, including six articles that assessed all four SNAP risk factors [21,22,23,24,25,26]. The most commonly assessed risk factor was smoking (*n* = 46, 74%), followed by physical activity (*n* = 33, 53%) and/or nutrition/diet (*n* = 17, 27%).

#### 3.1.3. Risk Assessment Tools/Models/Measures

A total of 21 distinct CVD risk assessment tools were identified from the 62 articles; six of these did not require clinical or laboratory data in their estimation (i.e., non-clinical).

The non-clinically based tools were mostly questionnaires or health surveys and included the Heart Disease Fact Questionnaire (HDFQ), the Health Beliefs Related to CVD-Perception measure (HBCVD), the Healthy Eating Opinion Survey, the Perception of Risk of Heart Disease Scale (PRHDS) and the WHO STEPwise approach to chronic disease factor surveillance (i.e., the STEPS instrument). The HDFQ is a validated and reliable 25 true/false-item questionnaire developed to assess heart disease knowledge among individuals with diabetes [43]. Each questionnaire item is a specific recommendation from at least one of three organizations—the American Diabetes Association, the American Heart Association, and the National Diabetes Education Program. Since the HDFQ assesses heart disease knowledge in people with diabetes, it is heavily skewed on diabetes-related CHD risk factors. Thus, further testing of its predictive validity is required for other health behaviors such as healthy eating, self-monitoring of blood glucose, or CHD diagnosis [43].

The HBCVD has been used to assess the perceptions of cardiovascular risk factors among individuals with type 2 diabetes. This is a 25 item questionnaire that assesses four constructs of the Health Belief Model (HBM), namely perceived susceptibility and severity of CVD, and benefits and barriers to diet and exercise [44]. However, further reliability testing of this tool is proposed.

The Healthy Eating Opinion Survey is a 43 item questionnaire assessing the psychosocial influences on dietary behavior in individuals at risk for CHD [31]. It was developed based on the Theory of Planned Behavior and assesses one’s intention (5 items), attitude toward the behavior (6 items), subjective norm (6 items), perceived behavioral control (5 items), behavior belief (10 items), normative belief (5 items), and control belief (6 items).

The PRHDS is a 20 item instrument developed to measure an individual’s perception of his/her heart disease risk in three dimensions—“dread risk”, (perceived lack of control, dread, catastrophic potential, and fatal consequences) “risk”, (a hazard with few, moderate, known outcomes and consequences) and “unknown risk” (hazards judged to be observable, unknown, new, and delayed in their manifestation of harm) [42].

The STEPS instrument was developed by the WHO for collecting data and measuring non-communicable disease (NCD) risk factors in three sequential levels, or “steps”—questionnaire, physical, and biochemical measurements [45]. It includes a core, an expanded, and optional components that provide a framework for countries conducting NCD risk factor surveys and allows each country to choose which of the three steps it will implement [45]. Steps 1 and 2 require non-clinical data, whereas step 3 depends on clinical data; thus, the STEPS instrument could be used as either a clinical- or non-clinical risk assessment tool.

The identified clinically based tools were the 10 year and 30 year Framingham Risk Score (FRS), Atherosclerotic CVD (ASCVD) risk calculator, Pathobiological determinants of atherosclerosis in youth (PDAY) risk score, Korean coronary CHD risk score, HellenicSCORE, AHA Ideal Cardiovascular Health (IDEAL) metrics, Progetto CUORE equation, Framingham CHD Prediction Score tool, HeartScore, Framingham risk equations (Joint British Societies 2 [JBS2] risk calculator), and the Systematic Coronary Risk Evaluation (SCORE). The FRS was the most commonly used CVD risk assessment tool in assessments of the young adult population.

#### 3.1.4. Sample Size

The number of participants in each of the included studies ranged from 15 to 619,130 (median = 2000).

### 3.2. Studies Assessing CVD Risks in the Underserved Young Adult Populations

Only five articles were related to CVD risk assessment in underserved young adult populations, and these originated in the U.S. (Table 4).

#### 3.2.1. Study Designs and Populations

The included articles comprised two cross-sectional studies, two longitudinal studies, and a qualitative (descriptive) study. Except for the qualitative study, the study population fell within the targeted age range (18–34 years, Table 4). Regarding the study populations, all but one study used data from national surveys.

#### 3.2.2. CVD Outcomes

The assessed CVD outcomes included the prevalence of CVD risk factors, cardio-metabolic disease risk, the perception of CVD risk, and history of CHD risk factors. Only one [43] out of the five articles assessed all four SNAP risk factors (Table 4).

#### 3.2.3. Risk Assessment Tools/Models/Measures

The 30 year Framingham CVD Risk score was the only identified clinically based CVD risk assessment tool. Non-clinically based tools were mostly surveys and questionnaires.

#### 3.2.4. Sample Size

The number of participants in each of the included studies ranged from 10 to 121,284 (median = 6052).

## 4. Discussion

This systematic literature review aimed to critically appraise, compare, and summarize existing non-clinically based tools for assessing CVD risk factors in young adult populations, particularly underserved young adults. Results showed that most risk assessment tools used in the young adult population were clinically based and included what have been and are still used in middle-aged and older adults, with the FRS tool being the most common one. Additionally, a modified version of the FRS, the 30 year FRS tool, was identified as an assessment tool in a study involving underserved young adults [46]. Unlike the original 10 year FRS, the 30 year FRS tool predicts an individual’s risk of developing CVD within 30 years and was specifically designed to be used in the young adult population [50].

The non-clinically based tools identified differed from the five non-laboratory-based cardiovascular risk assessment algorithms identified in a previous review [51]: the Framingham non-laboratory-based method, the Gaziano non-laboratory-based algorithm, the WHO/International Society of Hypertension (WHO/ISH) non-laboratory-based algorithms, the Swedish consultation-based method, and the United Kingdom (UK) General Practice model. The Framingham non-laboratory-based algorithm uses office-based predictors that are obtained in primary care (i.e., age, body mass index (BMI), systolic blood pressure, antihypertensive medication use, current smoking, and diabetes status) to predict 10 year CVD risk [52]. The Gaziano non-laboratory-based algorithm predicts CVD events using age, sex, smoking, diabetes, systolic blood pressure, antihypertensive medication use, and BMI [53]. The WHO/ISH algorithms predict 10-year cardiovascular risk using easily measurable variables, such as gender, systolic blood pressure, smoking status, type 2 diabetes mellitus, and total serum cholesterol [54]. The Swedish consultation-based method predicts cardiovascular risk using age, sex, current smoking, prevalence of diabetes or hypertension at baseline, blood pressure, waist-to-height ratio, and family history of CVD. The UK General Practice model uses age, systolic blood pressure, smoking habit, and self-rated health to predict 10 year CVD risk in older women [55].

Unlike previously identified tools, the non-clinically based tools identified in this review either assessed an individual’s knowledge or perception of heart disease risk. They did not directly assess one’s CVD risk in relation to the four SNAP risk factors.

The timely identification of young adults at high risk for CVD will help to reduce risk factor burden [56]. However, the selected age range in this review (i.e., 18–34 years) differs from that of the samples used in previous studies. For example, Alssema et al. [9] used a sample of adults aged 28–85 years to develop a single non-laboratory-based model for predicting three cardio-metabolic diseases, CVD, type 2 diabetes, or chronic kidney disease, in three different population cohorts. Additionally, in the study by Jamil et al. [41], the 18–39 years age group was the least represented; thus, the authors suggested using a relatively younger sample to make the findings more generalizable.

Furthermore, the five previously mentioned non-laboratory-based risk assessment tools relied on varied samples of middle-aged and older adults [51]. For instance, the Framingham non-laboratory-based algorithm was derived from an adult sample aged 30–74 years [52]; the Gaziano non-laboratory-based algorithm used an ethnically and racially diverse sample of 25–74-year-old adults [18,53]; the Swedish consultation-based method was derived from a sample of Swedish adults aged 40–59 years; and the UK General Practice model used only women aged 60–79 years [54]. Further validation of these non-laboratory-based tools in diverse populations is recommended to improve their performance and applicability in the screening and management of CVD in limited-resource settings [51].

Considering that the non-clinical risk assessment tools identified in this review were developed to assess CVD risks, none assessed all four SNAP risk factors together. The knowledge assessed with HDFQ pertained to smoking, healthy eating, and physical activity in relation to heart disease, as well as the relationship between diabetes and heart disease. Unlike the HDFQ, the HBCVD and the PRHDS do not assess a specific health behavior in relation to CVD risk, but an individual’s health beliefs in a likely CVD event and CVD risk perceptions, respectively. It was not surprising that smoking was the most commonly assessed SNAP risk factor in the included studies, considering most of the existing CVD risk scores use an individual’s smoking status as a predictor in their calculations [14,16]. An individual’s knowledge and perception may provide some useful information about an individual’s behavior but may not necessarily predict their CVD risk. Thus, incorporating all four SNAP risk factors in a non-clinically based CVD risk assessment tool may provide a broader picture of disease risk.

### Strengths and Limitations

A major strength of the present review is that it used a concept table in combination with a previously used search strategy that was thorough enough to identify the existing tools in adult populations. To the best of our knowledge, this is the first systematic review to identify tools or instruments that have been used to assess CVD risk factors in the young adult population. However, there are a few limitations. The number of articles excluded due to full-text unavailability might have caused an indirect omission of relevant details, especially from studies published in other languages. The grey literature was not searched even though it could have also been a useful source of other non-laboratory-based risk assessment tools.

## 5. Conclusions

This review provides a summary of non-clinically based CVD risk assessment tools used in the general young adults and underserved young adult populations. The findings indicated that generally, there were only a few objective and/or self-reported measure(s) for the non-clinical assessment of modifiable CVD risk factors among young adults. The main non-clinically based tools identified were the Heart Disease Fact Questionnaire, the Health Beliefs Related to CVD-Perception measure, the Healthy Eating Opinion Survey, the Perception of Risk of Heart Disease Scale, and the WHO STEPwise approach to chronic disease factor surveillance (i.e., the STEPS instrument). The identified tools assessed individuals’ knowledge or perception of heart disease risk but did not directly assess their CVD risk in relation to the four SNAP risk factors (i.e., smoking, nutrition behaviors, alcohol use, and physical activity). Future studies could adapt items from the identified non-clinically based CVD risk assessment tools, incorporating the four SNAP risk factors to develop a non-clinically based risk assessment tool, and validate it in young adults.

## Figures and Tables

**Table 1:** Concept table for the literature search.

	Cardiovascular Disease	Risk Assessment	Tool	Young Adult	Vulnerable Populations
MeSH terms/subheadings	Cardiovascular Diseases	Risk Assessment	Surveys and questionnaires, patient-reported outcome measures, healthcare surveys	Young Adult	Vulnerable populations, medically underserved area
Text words	cardiovascular AND diseases, “cardiovascular diseases”, cardiovascular AND disease OR “cardiovascular disease” heart diseases, heart disease, cerebrovascular diseases, hypertension, myocardial ischemia, myocardial infarction, heart attack, cardiovascular stroke, cerebral hemorrhage, cerebral stroke, stroke, brain ischemia	Risk Assessments, Health Risk Assessment, Health Risk Assessments, Risk Factors, Risk Prediction, Risk Models, Risk Prediction Models	Tools, instrumentation, instruments, community surveys, surveys, questionnaires, “surveys and questionnaires,” measures, outcomes assessment, outcome measures	young adult, young adults	Disadvantaged, Underserved Patients, Underserved Populations, Sensitive Population Groups, Sensitive Populations, Medically Underserved Population, vulnerable, limited [All Fields] AND health resources [mh]

**Table 2:** Inclusion and exclusion criteria of the review.

Component	Included	Excluded
Participants	Specified age range of study participants fell within the target range of the current study (i.e., 18–34 years) or if analyses were subdivided by age groups. Underserved young adults (18–34 years).	Studies that did not specify any age range for participants but only stated a median or mean age were excluded because it was difficult to ascertain which age groups were being discussed. Studies that recruited only participants from groups with diagnosed conditions linked to the SNAP risk factors (e.g., type 2 diabetes, hypertension) or from specific/special populations (e.g., severe mental illness, eating disorders, elite athletes) as this review was to help select an appropriate tool (i.e., non-clinically based) for assessing CVD risk factors in both symptomatic and asymptomatic young adults.
Intervention	Studies designed to increase CVD risk awareness or prevent CVD by altering one or more SNAP risk factors or at least one CVD outcome.	
Comparators	Any comparators/controls	
Outcome	The main outcome was objective and/or self-reported measure(s) for the non-clinical assessment of modifiable CVD risk factors by evaluating the SNAP risk factors. Studies that: (a) developed and/or used a questionnaire or instrument to assess at least one CVD outcome (b) developed and validated any tool to assess at least two SNAP risk factors in young adults or underserved young adults (c) reported on CVD risk assessment and/or treatment in people without prior CVD, or in people with and without prior CVD where this information is presented separately (d) reported on all measures developed and/or used in health promotion studies that aimed to increase CVD risk awareness or prevent CVD by altering one or more SNAP risk factors	Articles without full text.
Study design	Observational, experimental, and trial studies.	Review/meta-analyses

**Table 3:** Summary of included studies that used non-clinical tools for risk assessment in the general young adult population.

No.	Author(s); Year of Publication	Study Population	Country	Sample Size	Age (Years)	Gender	Modifiable CVD Risk Factors Assessed (Smoking, Nutrition/Diet, Alcohol Use, or Physical Activity)	Risk Assessment Measure/Tool
1	Williamson W et al., 2018 [27]	Young adults without clinical evidence of cerebrovascular disease	U.K	125	18–40	49% female	Smoking, alcohol use, physical activity	Detailed questionnaire on medical history, socioeconomic status, and self-reported behaviors such as nutritional intake, smoking, and alcohol consumption.
2	Tran D-T et al., 2016 [28]	College students at a Midwestern institution	U.S.A.	100	19–39	Male & female *	None	Heart Disease Fact Questionnaire; The Health Beliefs Related to Cardiovascular Disease
3	Thorpe RJ et al., 2016 [29]	Participants from 2000–2009 National Health Interview Surveys	U.S.A.	619,130	18–75+	52.1% female	Physical activity	Health survey
4	Lai HL et al., 2015 [30]	East Carolina University undergraduates	U.S.A	525	16–23	60.7% female	Smoking, physical activity	Health survey (internally validated)
5	Mark AE et al., 2014 [31]	Individuals at risk for coronary heart disease	U.S.A.	388	22–78	60.6% female	Nutrition/diet	Questionnaire (the Healthy Eating Opinion Survey)
6	Bloomfield GS et al., 2013 [21]	Adults [Health and Demographic Surveillance System]	Kenya	4037	18–>64	61% female	Smoking, nutrition/diet, alcohol use, physical activity	Home-based survey using the WHO STEPwise approach to chronic disease risk factor surveillance (WHO STEPS)
7	Schmitz R et al., 2012 [32]	Non-institutionalized adult population (National health interview [GEDA 2009] respondents.	Germany	21,262	18–≥65	51.5% female	Nutrition/diet, physical activity	Self-reported physician-diagnosed disease
8	Koura MR et al., 2012 [33]	Young adult females	Saudi Arabia	370	Mean = 19.9 ± 1.4	100% female	Smoking, nutrition/diet, physical activity	WHO-STEPS
9	Baragou S et al., 2012 [23]	The general adult population	Togo	2000	18–98	55.1% female	Smoking, nutrition/diet, alcohol use, physical activity	WHO STEPS
10	Foulds HJA et al., 2012 [34]	Aboriginal adult population (participants from the Hearts in Training and Health Beat physical activity training programs)	Canada	882	16–77	75.2% female	Smoking, physical activity	Multiple-choice questions
11	Chan CW et al., 2012 [35]	Hong Kong Chinese population	Hong Kong	236	18–91	66.5% female	None	Survey
12	Maniadakis N et al., 2011 [36]	General adult population	Greece	3007	18–>65	51.7% female	None	Survey
13	Al Hamarneh YN et al., 2011 [25]	General adult population	Northern Ireland	1000	20–79	46% females	Smoking, nutrition/diet, alcohol use, physical activity	Questionnaire
14	Kuklina EV et al., 2010 [37]	Participants from the National Health and Nutrition Examination Survey (NHANES)	U.S.A.	2587	20–35 (male); 20–45 (female)	61.2% female	Smoking	Survey
15	Wamala JF et al., 2009 [38]	Adult population	Uganda	842	20–>75	48% female	Smoking, alcohol use, physical activity	Questionnaire
16	Bjartveit K et al., 2009 [39]	Individuals surveyed for CVD risk factors	Norway	48,682	20–49	51.6% female	Smoking, physical activity	Questionnaire
17	Tucker AM et al., 2009 [40]	Veteran football players	U.S.A.	504	23–35	100% male	Smoking	Survey instrument
18	Sanderson SC et al., 2009 [26]	Respondents from the Office of National Statistics Omnibus Survey	U.K.	1747	16–75	47% female	Smoking, nutrition/diet, alcohol use, physical activity	Questionnaire
19	Jamil H et al., 2009 [41]	Respondents from the Health Assessment Survey	U.S.A.	3280	18–75	71.9% female	Smoking, nutrition/diet, physical activity	Health survey
20	Ammouri AA et al., 2008 [42]	General population	Jordan	295	15–75	51% female	None	Questionnaire (The Perception of Risk of Heart Disease Scale)

* Gender distribution not stated in article. (Note: A survey is a method of data collection and analysis, whereas a questionnaire is a tool or instrument used to collect data; a questionnaire may be a subset of a survey).

**Table 4:** Summary of included studies that assessed CVD risk in underserved young adult populations.

No.	Author(s); Year of Publication	Study Population	Country	Sample Size	Age (Years)	Gender	Modifiable CVD Risk Factors Assessed (Smoking, Nutrition/Diet, Alcohol Use, or Physical Activity)	Risk Assessment Measure/Tool
1	Doom JR et al., 2017 [46]	Add Health study participants	U.S.A.	14,493	24–34	48.9% female	Smoking, nutrition/diet, alcohol use, physical activity	30 year Framingham CVD Risk Score
2	Abshire DA et al., 2016 [47]	Undergraduate Caucasian males recruited from a public, 4 year university through purposive and snowball sampling; free of CVD and not enrolled in a health-related major.	U.S.A.	10	18–25	100% male	None	Interview guide
3	Wickrama KAS et al., 2016 [48]	Add Health study participants	U.S.A.	8824	24–32	Male & female *	None	None; biomarkers assessed
4	Khan RJ et al., 2015 [49]	1997–2004 data from National Health Interview Survey	U.S.A.	121,284	18–44	54.5% female	Smoking, physical activity	None
5	Jamil H et al., 2009 [41]	Respondents from the Health Assessment Survey	U.S.A.	3280	18–75	71.9% female	Smoking, nutrition/diet, physical activity	Health survey

* Gender distribution not stated in the article.

## Data Availability

No new data were created or analyzed in this study. Data sharing is not applicable to this article.

## Supplementary Materials

The following are available online at https://www.mdpi.com/article/10.3390/ijerph182413305/s1, Table S1: Search strategy used in Pubmed, Figure S1: Sample of data extraction form used.
