# Supplementary material for: Tools for Assessing Cardiovascular Disease Risk Factors in Underserved Young Adult Populations: A Systematic Review
Source: Int J Environ Res Public Health. 2021 Dec 17;18(24):13305. doi: 10.3390/ijerph182413305 (PMC8707965; doi:10.3390/ijerph182413305)
Supplement: Supplementary file 1 [file ijerph-18-13305-s001.zip › ijerph-1468401-supplementary.pdf]

Table S1. Search strategy used in PubMed

	Cardiovascular Diseases	
1	cardiovascular disease*[tiab]	158,869
2	cardiovascular diseases[majr:noexp]	95,167
3	cardiovascular diseases[mh]	2,256,281
4	cardiovascular diseases[mh:noexp]	136,098
5	myocardial infarction[majr]	124,856
6	coronary disease[majr]	163,987
7	stroke[majr]	89,288
8	brain ischemia[majr:noexp]	37,147
9	cerebrovascular accident[tiab]	4,211
10	death, sudden, cardiac[majr]	9,124
11	heart diseases[majr:noexp]	48,891
12	cardiovascular mortality[tiab]	12,184
13	coronary[tiab]	378,233
14	artery[tiab]	487,812
15	disease[tiab]	2,867,168
16	*(#13) AND (#14 OR #15)*	*263,475*
17	stroke[tiab]	218,939
18	brain[tiab]	929,305
19	cerebrovascular[tiab]	53,207
20	cerebral[tiab]	344,308
21	brainstem[tiab]	43,444
22	*(#17) AND (#18 OR #19 OR #20 OR #21)*	*68,560*
23	intracranial hemorrhages[majr]	47,765
24	intracranial hemorrhage, traumatic[majr]	9,136
25	cerebral hemorrhages[majr]	21,291
26	*((#23 NOT #24)) OR #25*	*39,587*
27	*(#1 OR #2 OR #4 OR #5 OR #6 OR #7 OR #8 OR #9 OR #10 OR #11 OR #12 OR #16 OR #22 OR #26)*	*850,063*
	Risk Assessment	
28	risk assessment[mh]	242,173
29	Risk Assess*[tiab]	60,378
30	Health Risk Assess*[tiab]	3,539
31	Risk Function*[tiab]	607
32	Risk Equation*[tiab]	583
33	Risk Calc*[tiab]	1,981
34	Risk Scor*[tiab]	17,789
35	Risk Predict*[tiab]	9,746
36	Risk Factor Calc*[tiab]	3
37	Risk Factor Assess*[tiab]	785
38	Risk Chart*[tiab]	223
39	Risk Engine*[tiab]	161
40	Risk Appraisal*[tiab]	652
41	Prediction Model*[tiab]	14,171
42	Risk algorithm[tiab]	233

43	Scoring* Method*[tiab]	43,976
44	Scoring Scheme*[tiab]	789
45	*(#28 OR #29 OR #30 OR #31 OR #32 OR #33 OR #34 OR #35 OR #36 OR #37 OR #38 OR #39 OR #40 OR #41 OR #42 OR #43 OR #44)*	*346,361*
	Coronary Artery Disease Risk Models	
46	assign score*[tiab]	50
47	brhs[tiab]	26
48	British regional heart[tiab]	143
49	British national heart[tiab]	52
50	busselton[tiab]	247
51	decode study[tiab]	27
52	Dundee risk score*[tiab]	4
53	erica risk[tiab]	229
54	findris*[tiab]	165
55	framingham equation*[tiab]	205
56	framingham estim*[tiab]	8
57	framingham heart study algorithm[tiab]	2
58	Framingham algorithm[tiab]	49
59	Framingham guideline*[tiab]	607
60	Framingham risk[tiab]	2,622
61	Framingham score*[tiab]	402
62	Framingham function*[tiab]	52
63	Framingham model*[tiab]	98
64	Glostrup[tiab]	327
65	New Zealand chart*[tiab]	515
66	precard[tiab]	5
67	PROCAM[tiab]	233
68	Reynolds risk score*[tiab]	68
69	score project[tiab]	56
70	Sheffield table*[tiab]	34
71	shaper score*[tiab]	21
72	Systematic Coronary Risk Evaluation[tiab]	296
73	*(#46 OR #47 OR #48 OR #49 OR #50 OR #51 OR #52 OR #53 OR #54 OR #55 OR #56 OR #57 OR #58 OR #59 OR #60 OR #61 OR #62 OR #63 OR #64 OR #65 OR #66 OR #67 OR #68 OR #69 OR #70 OR #71 OR #72)*	*5,706*
	Tools	
74	(surveys and questionnaires[majr:noexp])	*42,079*
75	patient reported outcome measures[majr:noexp]	1,630
76	health care surveys[majr:noexp]	3,808
77	survey tools[tiab]	201
78	survey instrument*[tiab]	3,760
79	community surveys[majr:noexp]	*42,079*
80	("surveys and questionnaires"[tiab])	889
81	(#74 NOT #80)	42,027
82	outcome measure*[tiab]	207,525
83	outcome assessments[tiab]	1,121

84	*(#75 OR #76 OR #77 OR #78 OR #81 OR #82 OR #83)*	*256,161*
	Underserved populations	
85	vulnerable populations[majr:noexp]	4,132
86	medically underserved area*[majr:noexp]	3,189
87	disadvantaged populations[tiab]	851
88	(underserved[tiab]) AND (patients OR populations[tiab])	4,624
89	sensitive population*[tiab]	*537*
90	sensitive population group*[tiab]	17
91	medically underserved population*[tiab]	246
92	(#86 OR #91)	3,377
93	(#89 OR #90)	*537*
94	(limited) AND health resource*[mh]	1,670
95	*(#85 OR #87 OR #88 OR #92 OR #93 OR #94)*	*14,124*
	Summation	
96	*(#45 AND #84 AND #73)*	*149*
97	(#27 AND #96 AND English[la] AND humans[mh] AND young adult*[mh] AND 2008:2019[dp])	5
98	(#27 AND #96 AND English[la] AND humans[mh] AND #95 AND 2008:2019[dp])	0
99	(#27 AND #96 AND English[la] AND humans[mh] AND young adult*[mh] AND #95 AND 2008:2019[dp])	0
100	*(#27 AND #96 AND English[la] AND humans[mh] OR young adult*[mh] AND #95 AND 2008:2019[dp])*	*1,126*
101	(#27 AND #96 AND English[la] AND humans[mh] AND young adult*[tiab] AND 2008:2019[dp])	1
102	*(#27 AND #96 AND English[la] AND humans[mh] OR young adult*[tiab] AND #95 AND 2008:2019[dp])*	*59*
103	*(#45 OR #84 OR #73)*	*594,736*
104	(#27 AND #103 AND English[la] AND humans[mh] AND young adult*[mh] AND 2008:2019[dp])	2,934
105	(#27 AND #103 AND English[la] AND humans[mh] AND #95 AND 2008:2019[dp])	43
106	(#27 AND #103 AND English[la] AND humans[mh] AND young adult*[mh] AND #95 AND 2008:2019[dp])	2
107	*(#27 AND #103 AND English[la] AND humans[mh] OR young adult*[mh] AND #95 AND 2008:2019[dp])*	*1,167*
108	(#27 AND #103 AND English[la] AND humans[mh] AND young adult*[tiab] AND 2008:2019[dp])	365
109	*(#27 AND #103 AND English[la] AND humans[mh] OR young adult*[tiab] AND #95 AND 2008:2019[dp])*	102
110	*((#45 AND #84)) OR #73*	*15,915*
111	(#27 AND #110 AND English[la] AND humans[mh] AND young adult*[mh] AND 2008:2019[dp])	298
112	(#27 AND #110 AND English[la] AND humans[mh] AND #95 AND 2008:2019[dp])	6
113	(#27 AND #110 AND English[la] AND humans[mh] AND young adult*[mh] AND #95 AND 2008:2019[dp])	0

114	*(#27 AND #110 AND English[la] AND humans[mh] OR young adult*[mh] AND #95 AND 2008:2019[dp])*	1,132
115	(#27 AND #110 AND English[la] AND humans[mh] AND young adult*[tiab] AND 2008:2019[dp])	33
116	*(#27 AND #110 AND English[la] AND humans[mh] OR young adult*[tiab] AND #95 AND 2008:2019[dp])*	65
	Tools in young adults (SR research question 1)	
117	#97 OR #101 OR #104 OR #108 OR #111 OR #115	3,108
118	#117 AND letter[pt]	23
119	#117 AND comment[pt]	17
120	#117 AND editorial[pt]	14
121	#118 OR #119 OR #120	42
122	#117 NOT #121	3,066 (3,110 as at 5/28/19; 3,121 as at 5/29/19)
	Tools for underserved young adults (SR research question 2)	
123	#100 OR #102 OR #105 OR #106 OR #107 OR #109 OR #112 OR #114 OR #116	1,188
124	#123 AND letter[pt]	5
125	#123 AND comment[pt]	0
126	#123 AND editorial[pt]	2
127	#124 OR #125 OR #126	7
128	#123 NOT #127	1,181

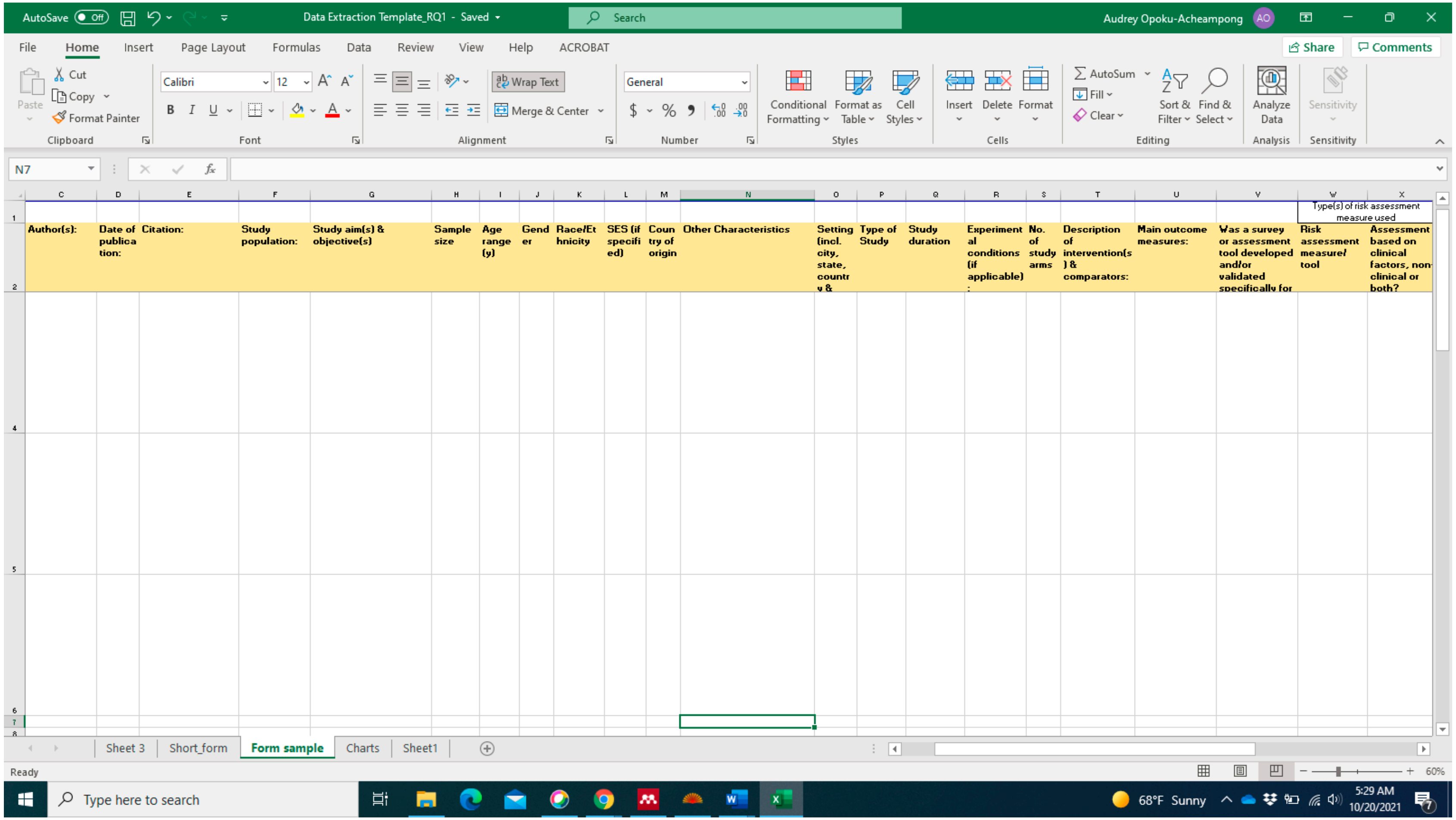

Figure S1. Sample of data extraction form used.
